# The impact of teacher preparation on preservice teachers' attitudes toward inclusive education in Qatar

**DOI:** 10.1016/j.heliyon.2021.e07925

**Published:** 2021-09-02

**Authors:** Elsayed E.A. Hassanein, Yousef M. Alshaboul, Sayed Ibrahim

**Affiliations:** Qatar University, Doha, Qatar

**Keywords:** Inclusive education, Disability, Attitude change, Preservice teachers, Teacher training, Qatar

## Abstract

Teachers' beliefs and attitudes are a significant component in the development and success of inclusive education. Research indicates that the foundation of positive attitudes toward inclusive education can be established in preservice-teacher-preparation programs. This study examines the change in preservice teachers' attitudes toward inclusive education following an inclusion-centered course, combined with an 18-hr practicum. Using the Multidimensional Attitudes toward Inclusive Education Scale (MATIES), 98 preservice teachers from primary and secondary teacher education programs in the College of Education at Qatar University in Doha, Qatar, were surveyed pre and post-course. It was found that all the participants' attitudes toward inclusive education changed significantly. No significant differences between primary and secondary preservice teachers were found at the end of the course. The results illustrated that combining information-based instruction with structured fieldwork experiences can potentially change preservice teachers' attitudes toward inclusive education. Implications for practice and future directions in research are considered.

## 1 Introduction

In the last three decades, there has been a significant movement toward inclusive education worldwide. Many declarations enhanced this movement from different United Nations agencies that encouraged countries worldwide to reform educational policies to promote inclusive education (e.g., Salamanca Statement, UNESCO, 1994, Convention on the Rights of Persons with Disabilities (CRPD), United Nations, 2006). Qatar signed and ratified the CRPD in 2007, thereby obligating itself to eliminate discrimination against children with disabilities and make their inclusion into the educational system a priority. Furthermore, Qatar has issued a set of laws and modern legislations and policies aimed at protecting and promoting the rights of persons with disabilities to ensure the right of access to the maximum independence that enables them to their potential and enables them to participate actively in society (Al-Attiyah and Hassanein, 2017).

For example, in 2004, Qatar issued Law No. (2), the first Qatari law to regulate the rights of individuals with disabilities. It includes the concept of people with disabilities, rehabilitation, care, and the support services that should be provided to individuals with disabilities and their rights. Additionally, Law No. (2) included the right to education and rehabilitation for individuals with disabilities, each according to his capabilities, with the need to provide appropriate educational and rehabilitation programs and prepare qualified cadres to deal with them.

### 1.1 Context of the study

Regarding the responsibility for establishing and implementing policies and plans for students with disabilities, in 2002, Qatar's Supreme Education Council (SEC) directed two administrative bodies, the Education Institute, and the Additional Educational Support Needs (AESN) department. The purpose of the AESN was to support schools' use of teaching and learning best practices to meet students' with disabilities needs in the Qatari independent schools. Qatar adopted the term “additional educational support needs” to identify students with disabilities and special needs attending independent schools. Independent schools and the AESN were directed to provide all classrooms with student services and support (SEC, 2009).

The Education Institute oversees independent schools and assists them in designing educational plans that identify the individual needs of each student. The Institute helps independent schools (currently public schools) by preparing educational policies for the schools and providing help for students with additional educational support needs (SEC, 2009). The Education Institute adopted the Response to Intervention (RTI) model to meet the needs of students with disabilities. Based on research, the RTI model is utilized to develop and support continuation plans. RTI components include a screening process, progress monitoring and reporting, and a prevention system that works at multiple levels. Al-Attiyah and Lazarus (2012) note that all RTI components are currently implemented in general education classrooms in Qatar schools.

The RTI model includes three levels of intervention (SEC, 2009). The first level of support begins when a student does not make expected progress in the classroom. The interventions are designed to be preventative and are started as soon as progress monitoring identifies a student who is falling behind and not acquiring academic skills (such as reading). There must be frequent, relevant data collection at this level.

The second level (level two) of RTI support focuses on following a multidisciplinary collaborative problem-solving process to come up with teaching methods to help students who need more support with the educational program of the classroom. The methods should be empirically based intervention strategies that use available resources in the school, home, and community to implement and sustain these interventions. There must be systematic measuring of the students' progress with these interventions. If the student still has problems, then level three is considered for more intensive support (VanDerHeyden & Jimerson, 2005).

The third level (level three) of intervention requires more specialized intensive teaching methods. It may be necessary to consider the student's placement outside the general classroom to meet their needs. The level three assessment would be a comprehensive multidisciplinary assessment of the student's educational needs.

The RTI model focuses on improving teaching and assessment procedures to meet the needs of all children in the classroom. Improvements may incorporate making modifications to the curriculum and assessments and generally includes an emphasis on using technology. The classroom program is presented and modified as needed by the teacher with the help of the additional educational support coordinator.

The Supreme Educational Council (SEC) (currently the Ministry of Education and Higher Education) is committed to providing a comprehensive education service that meets all students' needs and provides them with the highest quality of learning experiences. Policies and guidelines have been prepared to help and support schools to meet the ethical, educational, and legal requirements of Education for All (SEC, 2009).

Despite all these efforts to enhance inclusive education in Qatar, Al-Attiyah (2012) found that teachers have some concerns about inclusive education and do not fully support inclusive education. Furthermore, teachers still opt for segregated settings in the education of children with disabilities as they preferred the educational settings in the following order: a classroom attached to the regular school, a special education center, a traditional classroom with the provision of special education services, a resource room, a regular classroom. Similar concerns about inclusive education were reported in some other Arabic countries like Egypt (Hassanein et al., 2021), Jordan (Amr et al., 2016), United Arab Emirates (Gaad and Almotairi, 2013) and Lebanon (Khochen and Radford, 2012). Moreover, it is claimed that the majority of teachers in the Arab region tend to demonstrate negative attitudes towards inclusive education (Hadidi and Al Khateeb, 2015). Even if the government provides all the materialistic, luxurious requirements for inclusion, their efforts will be useless as long as the attitude of most teachers towards inclusion remains unchanged (Hadidi and Al Khateeb, 2015). It is claimed that teachers' concerns and negative attitudes could be adequately addressed in teacher training programs via providing well-designed courses about inclusion and diversity in teacher education programs.

The College of Education was the first higher education institution in Qatar and the founding unit of Qatar University. It remains the single entity for the preparation of educators in the country. The College of Education offers three undergraduate degree programs; primary education, secondary education, and special education. Additionally, the College of Education offers the following graduate degree programs: Diploma in Early Childhood Education, Diploma in Special Education, Diploma in Primary Education, Diploma in Secondary Education, Master of Arts in Curriculum, Instruction, and Assessment, Master of Education in Special Education and Master of Educational Leadership.

In 2002, as part of a widespread reform in education, all College of Education programs were closed or scheduled for closure so that new, more rigorous programs could be developed. In 2002, because of extreme national need, the Diploma in Special Education and a Diploma in Early Childhood were initiated, the first new programs added to the college, emphasizing the importance of this program to both the college and the country. In 2015, a Bachelor's degree in special education was initiated. Students in Diploma in Special Education and the Bachelor's degree in special education are in contact with several courses about disability, diversity, and inclusion. However, students in primary and secondary education programs study only one course about inclusive education. Although the country adopted inclusive education policies, teacher education programs offered in the college of education in Qatar University did not include any courses about inclusive education or diversity. It was until 2015 when the inclusive classrooms were provided for students in primary and secondary education programs. However, the quality of this course and its impact on teachers' skills and attitudes have not been investigated.

### 1.2 Theoretical framework

Hassanein (2010) contends that the academic literature presents different conceptualizations of attitudes, the most accepted models are the unidimensional and the multidimensional models. The unidimensional model considers attitudes as individuals' feelings and emotions expressed toward an attitude object, while the multidimensional model understands attitudes as tendencies to respond to stimuli with certain classes of responses (Eagly and Chaiken, 1993). These classes are identified as affective (regarding evaluative feelings of liking and disliking), cognitive (regarding beliefs, opinions, and ideas about the object of attitude), and behavioral (regarding the behavioral intentions or action tendencies).

The present study implemented the multidimensional model, which is associated with the theory of planned behavior (Ajzen, 1991, 2005). The theory of planned behavior (TPB) (Ajzen, 1991) affords a valuable framework to understand the relationship between attitude and behavior. TPB extends the theory of reasoned action (Ajzen and Fishbein, 1980) by attempting to predict certain behaviors, attitudes, subjective norms, perceived behavioral control, and behavioral intention about that behavior needed to be considered. “Subjective norm” is best defined as an individual's perception of how significant others will approve of their behavior. The individual's perception of the ease or difficulty to perform the behavior is considered the perceived behavioral control, while the willingness to perform any given behavior is the behavioral intention. TPB predicts that it is more likely for an individual to perform a behavior if each component is favorable. Specifically, TPB contends that behavioral intention is determined by attitudes, subjective norms, and perceived behavioral control. Behavior is determined by attitudes, subjective norms, and perceived behavioral control, mediated by behavioral intention. Moreover, attitudes could consist of cognitive (beliefs) and affective (feelings) dimensions (Ajzen, 1991; Eagly and Chaiken, 1993).

Aligned with TPB, this study contends that the development of intentions (within inclusive education) are influenced by (multidimensional) attitudes toward the behavior, perceived social pressure to perform or not perform a particular behavior (subjective norms), and the perceived ease or difficulty of performing the behavior (perceived behavioral control) reflected by previous experience and knowledge; and newly acquired knowledge (Ajzen, 1991). The individual's intention to perform the behavior is stronger if the attitudes and subjective norms concerning behavior are more favorable.

### 1.3 Teacher education and teachers' attitudes towards inclusive education

It is argued that, although inclusive education policies are in place, they neither guarantee successful experiences for students with disabilities (Peters et al., 2005; Yuknis, 2015) nor ensure that the policy is supported by those most accountable for effective implementation, specifically, classroom teachers (Campbell et al., 2003; Hassanein, 2015b). Research indicated that teachers' beliefs and attitudes are essential for the success of inclusive education since teachers' acceptance of this policy is expected to affect their commitment to implementing it (Beacham and Rouse, 2012; de Boer et al., 2011; Hassanein, 2015a; Saloviita and Schaffus, 2016). Additionally, research has reported that it is more likely for teachers who have positive attitudes toward inclusive education to adapt their teaching strategies to meet various learning needs (Berry, 2010; Blecker and Boakes, 2010; Sharma et al., 2008; Swain et al., 2012).

Furthermore, it is claimed that teachers often feel less prepared to deal with diversity in their classrooms (Forlin 2004; Hassanein et al., 2021). To address these concerns, Killoran et al. (2014) pointed out 'teacher-preparation programs must design courses that help prospective teachers appreciate environmental, social and cultural contexts of learning, behavior, and teaching, and be able to enact these understandings in inclusive classrooms serving increasingly diverse students' (p. 428). Moreover, several researchers argued that the foundation of positive and equitable attitudes toward inclusive education could be established in initial-teacher-preparation programs (Johnson and Howell, 2009; Jung, 2007; Killoran et al., 2014; Kim, 2011; Lambe, 2007; McHatton and Parker, 2013; Swain et al., 2012). Furthermore, research indicated that teachers who have positive attitudes toward inclusive education have mostly gone through preservice training, which addresses teachers' concerns and better prepares them to face the challenges that they may face in the field (Harvey et al., 2010).

However, research results on teacher-preparation programs and their effect on teachers' attitudes toward inclusive education are mixed (Lautenbach and Heyder, 2019; Swain et al., 2012). For example, Shade and Stewart (2001) found that an introductory special education course positively impacted the attitudes of prospective teachers' attitudes towards inclusion. Similarly, Shippen et al. (2005) found a statistically significant change in preservice teachers' attitudes toward inclusion at the end of an introductory course on exceptionality. Several other studies (e.g., Campbell et al., 2003; Johnson and Howell, 2009; Killoran et al., 2014; Taylor and Ringlaben, 2012) support that participating in such a course can positively affect preservice teachers' attitudes and beliefs about inclusive education.

However, Hodge et al. (2002) compared the effects of two practicum types on physical education teacher education students' attitudes toward teaching students with physical disabilities or moderate-severe mental retardation and found no significant change in their attitudes. Similarly, Kirk (1998) investigated the impact of university coursework on preservice teacher attitudes toward students with disabilities reporting that participating in this coursework had no impact on attitudes toward working with students with disabilities. Additionally, Yuknis (2015) found that an introductory special education course did not significantly impact preservice teachers' attitudes towards inclusion. Moreover, McHatton and Parker (2013) investigated elementary and special education preservice teachers' perceptions of inclusion following a classroom management course and a field training in K-5 classrooms. The results of this study showed statistically significant changes in the elementary preservice teachers, but no change in the special education preservice teachers.

### 1.4 Purpose of the current study

Considering these diverse results, more research is necessary to investigate what kind of program may be most effective to foster positive changes in preservice teachers' attitudes toward inclusive education. It is also important to research present teacher preparation programs and their effect on preservice teachers' attitudes, especially in contexts where inclusive education is still emerging, like Qatar. Moreover, while the large majority of previous studies in this area were conducted using Western participants (mostly Americans; see Lautenbach and Heyder, 2019), the present study was conducted using samples of preservice teachers from an Arab country (Qatar). To the researchers' best knowledge, this is the first study in the Gulf area, especially in Qatar.

Moreover, teachers live within a cultural context that must be considered in the research. It is, therefore, necessary to locate teachers' attitudes within a broader socio-cultural context adopting what Eiser (1994) called a “social constructivist” view of attitudes. In this view, attitudes are considered the product of the interaction between the personal and the social factors rather than a simple personal entity. Typically, cultures are separated into collectivist and individualist types. The majority of the research has been conducted in individualist countries such as the United States. Various studies have reported that there were more positive attitudes towards inclusion and individuals with disabilities in individualist societies than collectivist societies (e.g., Benomir et al., 2016; Hassanein, 2015a). Various factors could account for the variability in teachers' attitudes in collectivist societies. According to Hofstede's index, Qatar, which was the context for this study, is considered a highly collectivist society (Hofstede et al., 2010).

Furthermore, most of the previous research in this area investigated the impact of special education courses on preservice teachers' attitudes towards inclusive education. Such courses may not reflect the ethos of inclusive education precisely. Limited research investigated the importance of inclusion-centered courses on preservice teachers' attitudes towards inclusive education (Tournaki and Samuels, 2016). Therefore, the study reported in this manuscript contributes to the existing literature in this area.

Therefore, this study aimed at examining the change in preservice teachers' attitudes toward inclusive education following an inclusion-centered course (Inclusive Classrooms), combined with an 18-hour practicum experience observing and working with students with disabilities in a range of inclusive education settings. The study also examined differences in preservice teachers' attitudes according to their primary or secondary education major. Specifically, this study aims to answer the following questions:

• To what extent do student teachers' attitudes change over the course?
• To what extent do student teachers' attitudes differ according to their major, either primary or secondary education?

## 2 Method

### 2.1 Participants

The study employed a pre- and post-questionnaire design to compare the attitudes of all student teachers enrolled in the Inclusive classrooms course within the College of Education at the University of Qatar. College of Education at Qatar University offers a compulsory course about inclusive education (Inclusive Classrooms, EDUC317) to primary and secondary education students. A total of ninety-eight Female preservice education students (M = 22.9 years, SD = 3.4 years) were enrolled in this course in Spring 2019. There were no male students enrolled in this course at that time. All the students were invited to participate in the study. Of the 98 students enrolled in the course when the survey was implemented, 92 students responded to the pre-survey, post-survey, or both. Students who did not respond either to the pre- or post-survey (n = 6) were excluded from the analysis. Therefore, the responses of the 92 students (n = 54 primary and n = 38 secondary) who responded to both the pre- and post-inclusion survey were included in the analysis.

### 2.2 Measures

#### 2.2.1 Multidimensional Attitudes toward Inclusive Education Scale (MATIES)

The MATIES (Mahat, 2008) is an 18-item self-report measure assessing teachers' attitudes toward inclusive education. It consists of three domains: cognitive (6 items), affective (6 items), and behavioral (6 items) and uses a 6-point Likert scale ranging from 1 (strongly agree) to 6 (strongly disagree). Three of the items on the cognitive subscale and all items of the behavioral subscale were reverse coded. Reliability of the MATIES has been well-established, with internal consistency of the subscales as follows: cognitive, α = 0.77; affective, α = 0.78; and behavioral, α = 0.91 (Mahat, 2008). Additionally, the scale was validated and reviewed by seven experts knowledgeable in special education, inclusive education, and measurement (Mahat, 2008).

An Arabic translation for the MATIES has been validated and piloted on a sample of preservice teachers in Qatar (Hassanein, 2017). Internal consistency coefficients for the Arabic version are as follows: cognitive, α = 0.71; affective, α = 0.70; and behavioral, α = 0.78 (Hassanein, 2017). These high reliability rates were also replicated in the present study, with internal consistency coefficients as follows: cognitive, α = 0.72; affective, α = 0.70; and behavioral, α = 0.79.

#### 2.2.2 The introductory inclusion-centered course (inclusive classrooms)

This course was designed to prepare preservice teachers to effectively teach a range of students found in the general education classroom. Research-proven intervention techniques with children with different disabilities were addressed. Additionally, the special education litigations and policies, disability categories, medical and social models of disability, diversity, and behavior management were covered in this course. Furthermore, the course focused on improving classroom behavior and social skills, differentiation and effective instruction for all students, accommodations and modifications for students with disabilities in the general education classroom, assessment, and collaboration between regular and special education teachers. Preservice teachers enrolled in the course attended class three hours a week for 15 weeks, totaling 45 h of instruction. In addition to the inclusion-centered course, all preservice teachers participated in 18 h of fieldwork with students with disabilities in a range of inclusive education settings. Through this experience, the participants observed inclusive practices such as collaboration, accommodation, differentiation, and various behavior management techniques with a mentor teacher.

### 2.3 Procedures

Upon receiving Qatar University Institutional Review Board (QU-IRB) approval and permission from the instructor, preservice teachers were recruited. Informed consent was obtained from all participants. Ninety-two preservice teachers participated in the study (primary education n = 54 and secondary education n = 38). Participants completed the instrument twice, first during the first session of the course (pre-survey) and then during the last session (post-survey).

### 2.4 Data analysis

Means and standard deviations were computed for all of the items and subscales used in the current study. Changes in preservice teachers' attitudes were calculated using t-test, and the effect size was calculated using Cohen's d. All analyses were conducted using IBM SPSS v. 26.0.

## 3 Results

### 3.1 Changes in preservice teachers' attitudes

A paired sample t-test was conducted from pre-to post-survey to evaluate the changes in preservice teachers toward inclusive education at the end of the course. It was found that a significant change took place in the attitudes of preservice teachers on all subscales of attitudes scale (Cognitive t (91) = -11.65; p <. 001; affective t (91) = -13.96; p <. 001; and behavioral t (91) = -13.74; p <. 001). An examination of η2 values indicated that change in attitude mean scores were substantial for all subscales (cognitive = 1.21, affective = 1.45, and behavioral = 1.43). Values of η2 can range from 0.01 to 2.0, as initially suggested by Cohen (1988) and expanded by Sawilowsky (2009). According to Sawilowsky (2009) η2 values of 0.01, 0.20, 0.50, 080, 1.20, and 2.0 suggest a 'very small', 'small', 'medium', 'large', 'very large' and 'huge effect' respectively.

#### 3.1.1 Changes in preservice teachers' attitudes (cognitive component)

Individual items were analyzed from pre-to post-survey with statistically significant differences from pre-to post-survey found in all items (see Table 1). Across the cognitive subscale items, the most significant change in attitude scores was for Items six (I believe that students with a disability should be in special education schools so that they do not experience rejection in the regular school), and two (I believe that students with a disability should be taught in special education schools). By the end of the course, preservice teachers became more reluctant to place children with disabilities in segregated settings. An examination of η2 values indicated that change in attitude mean scores were 'medium', to 'large', for all the items of the cognitive subscale as the η2 values ranged between .40 and .95.

**Table 1:** Change in preservice teachers' attitudes towards inclusive education (cognitive component).

Item	*Pre-survey*		*Post-survey*		t	d
	*M*	*SD*	*M*	*SD*		
1. I believe that an inclusive school is one that permits academic progression of all students regardless of their ability	4.55	1.15	5.16	0.92	3.77∗∗	.40
2. I believe that students with a disability should be taught in special education schools.	3.38	1.54	4.70	1.23	7.49∗∗	.78
3. I believe that inclusion facilitates socially appropriate behavior amongst all students.	5.01	0.74	5.57	0.76	5.79∗∗	.60
4. I believe that any student can learn in the regular curriculum of the school if the curriculum is adapted to meet their individual needs.	4.64	0.97	5.32	1.02	5.41∗∗	.56
5. I believe that students with a disability should be segregated because it is too expensive to modify the physical environment of the school.	4.13	1.12	5.15	1.12	6.33∗∗	.66
6. I believe that students with a disability should be in special education schools so that they do not experience rejection in the regular school.	3.78	1.12	5.10	0.95	9.19∗∗	.95

∗∗ *p <* .01.

#### 3.1.2 Changes in preservice teachers' attitudes (affective component)

Across the affective subscale items, the most significant change in attitude scores was for Items ten (I am uncomfortable including students with a disability in a regular classroom with other students without a disability), and nine (I get irritated when I am unable to understand students with a disability) (see Table 2). Preservice teachers became more comfortable including students with a disability in a regular classroom by the end of the course. Additionally, an examination of η2 values indicated that change in attitude mean scores were 'medium', to ' large', for all the items of the affective subscale as the η2 values ranged between .60 and .96.

**Table 2:** Change in preservice teachers' attitudes towards inclusive education (affective component).

Item	*Presurvey*		*Postsurvey*		*t*	d
	*M*	*SD*	*M*	*SD*		
7. I get frustrated when I have difficulty communicating with students with a disability.	3.31	0.83	4.15	1.13	5.69∗∗	.60
8. I get upset when students with a disability cannot keep up with the day-to-day curriculum in my classroom.	2.95	0.94	3.70	0.76	6.12∗∗	.63
9. I get irritated when I am unable to understand students with a disability.	2.78	1.05	4.20	1.03	8.58∗∗	.90
10. I am uncomfortable including students with a disability in a regular classroom with other students without a disability.	4.26	0.76	5.38	0.93	9.21∗∗	.96
11. I am disconcerted that students with a disability are included in the regular classroom, regardless of the severity of the disability.	4.38	0.94	5.37	0.87	7.96∗∗	.83
12. I get frustrated when I have to adapt the curriculum to meet the individual needs of all students.	4.12	0.84	5.06	0.96	8.29∗∗	.86

∗∗ *p <* .01.

#### 3.1.3 Changes in preservice teachers' attitudes (behavioral component)

Across the behavioral subscale items, the most significant change in attitude scores was for Items 16 (I am willing to modify the physical environment to include students with a disability in the regular classroom) and 14 (I am eager to adapt the curriculum to meet the individual needs of all students regardless of their ability) (see Table 3). This reflects preservice teachers' intention to accommodate the needs of all children in terms of the physical environment and curriculum modification. Additionally, an examination of η2 values indicated that change in attitude mean scores were 'medium', to 'very large', for all the items of the behavioral subscale as the η2 values ranged between .32 and 1.36.

**Table 3:** Change in preservice teachers' attitudes towards inclusive education (behavioral component).

Item	*Presurvey*		*Postsurvey*		*t*	d
	*M*	*SD*	*M*	*SD*		
13. I am willing to encourage students with a disability to participate in all social activities in the regular classroom.	4.65	0.99	5.69	1.08	7.24∗∗	.75
14. I am willing to adapt the curriculum to meet the individual needs of all students regardless of their ability.	4.24	0.80	5.65	0.67	12.91∗∗	1.34
15. I am willing to physically include students with a severe disability in the regular classroom with the necessary support.	4.24	1.09	4.84	1.55	3.11∗∗	.32
16. I am willing to modify the physical environment to include students with a disability in the regular classroom.	4.55	0.59	5.67	0.59	13.04∗∗	1.36
17. I am willing to adapt my communication techniques to ensure that all students with an emotional and behavioral disorder can be successfully included in the regular classroom.	4.77	0.72	5.78	0.55	10.68∗∗	1.11
18. I am willing to adapt the assessment of individual students in order for inclusive education to take place.	4.68	0.70	5.80	0.61	11.06∗∗	1.15

∗∗ *p <* .01.

### 3.2 Differences in preservice teachers' attitudes according to their major (primary v secondary)

The baseline data (pre-survey) indicated no significant differences between primary and secondary education preservice teachers' attitudes toward inclusive education at the beginning of the course. The results of the independent sample t-test for the pre-survey attitude scores of primary and secondary education preservice teachers did not show any statistical difference (cognitive; *t* (90) = -.850; *p > .05*; affective; *t* (90) = 1.425; *p > .05*; and behavioral; *t* (90) = .752; *p > .05*). The means of the pre-survey scores for each of the subscales (cognitive, affective, and behavioral) of the primary education preservice teachers were 4.20; 3.68; 4.56, respectively, whereas secondary education preservice teachers had pre-survey means of 4.32; 3.57; 4.48 for the three subscales, respectively. The close mean of the participants' pre-survey attitude scores indicates that primary and secondary education preservice teachers had similar pre-survey attitude scores before the course.

Finally, an independent sample t-test was conducted to evaluate differences between primary and secondary education preservice teachers' attitudes toward inclusive education at the end of the course. The results of the Independent sample t-test for the post-survey attitudes scores of primary and secondary preservice teachers did not show any statistical difference (Cognitive t (90) = .394; p > .05; affective t (90) = .247; p > .05; and behavioral t (90) = .577; p > .05). The mean of the post-survey scores of the primary school preservice teachers was (5.18; 4.66; 5.60), whereas secondary preservice teachers had a presurvey mean of (5.13; 4.63; 5.54) for the three subscales, respectively. The close mean of the participants' post-survey attitude scores by the end of the course indicates that the introductory inclusion-centered course paired with field training effectively changed attitudes of both primary and secondary education preservice teachers toward inclusive education.

## 4 Discussion and conclusion

The study investigated the change in preservice teachers' attitudes toward inclusive education following an inclusion-centered course, combined with an 18-hour practicum experience. The findings supported the hypothesis that formal instruction with structured fieldwork experience fostered favorable changes in attitudes toward inclusive education.

Change in participants' attitudes, especially in the cognitive subscale that measures preservice teachers' ideas, thoughts, beliefs, or opinions about inclusive education, can be partially explained by the content and pedagogy of the course. During the course, research-proven intervention techniques with children with different disabilities were addressed. Such emphasis might have relieved preservice teachers' concerns about children with various disabilities and improved their attitudes towards including them. According to Sharma et al. (2008), focusing on the needs of children with different disabilities in academic courses could prepare prospective teachers to teach students with similar disabilities. Additionally, these results align with several previous studies (e.g., Campbell et al., 2003; Johnson and Howell, 2009; Killoran et al., 2014; Shade and Stewart, 2001; Shippen et al., 2005; Taylor and Ringlaben, 2012), which indicated that university information-based courses could lead to changes in preservice teachers' attitudes toward inclusion especially in issues related to beliefs and thoughts.

Two variables can explain the change in affective and behavioral attitudes toward inclusive education: course content and pedagogy and the other related to contact with persons with disabilities. The course's significant focus was on improving classroom behavior and social skills, differentiation and effective instruction for all students, and assessment and collaboration between regular and special education teachers. Such a focus might have built preservice teachers' confidence to teach children with different disabilities and accommodate them in their classes. This also might have relieved their concerns and made them less reluctant to include children with disabilities. This is in line with the results of Sharma et al. (2008), who claimed that an emphasis on effective teaching strategies might also have contributed to decreasing preservice teachers' concerns and fostered their positive attitudes toward inclusion.

Additionally, the changes in teacher's behavioral intentions could be attributed to the changes that happened in their beliefs and emotions represented in the first two components of attitudes. According to the multidimensional model of attitudes and the theory of planned behavior, change in preservice teachers' behavioral intentions (within inclusive education) may have been influenced by multidimensional attitudes toward the behavior (beliefs, subjective norms, and emotions). This indicates that the more favorable the attitudes and subjective norms concerning behavior, and the greater the perceived control behavior, the stronger the individual's intention to perform the behavior.

Moreover, change in preservice teachers' behavioral intentions could be attributed to the field experience which might have changed their assumptions and relieved their concerns about individuals with disabilities. This is based on Fazio and Zanna's (1981) suggestion that consistency between behavior and the affective component of attitude is likely to be higher for attitudes acquired through direct experience.

Furthermore, during fieldwork, students were exposed to persons with a disability. Preservice teachers spent considerable time observing and working with a student with a disability. This may have elevated their comfort level about interacting with persons with a disability and consequently changed their attitudes toward inclusive education. These results support the recommendations of Sharma et al. (2008) that carefully planned and supported contact with persons with disabilities can foster positive changes in teachers' attitudes toward inclusive education.

This study illustrated that combining information-based training programs with structured fieldwork positively changed attitudes toward inclusive education. These findings align with those of other research studies regarding the significance of structured fieldwork, in addition to university courses, in fostering attitude change toward inclusive education (e.g., Campbell et al., 2003; Gürsel, 2007; Johnson and Howell, 2009; Killoran et al., 2014; Lambe, 2007; Sharma et al., 2008; Swain et al., 2012).

The field experience linked to the inclusion-centered course offered preservice teachers opportunities to identify and rethink their assumptions about disability and inclusive education. The coursework blending with field experience enabled preservice teachers to understand their epistemological assumptions about disability and inclusion. This approach exemplifies a reflective practice model (Bayliss, 1998; Hassanein, 2015b) that allows the experience to be synthesized through theorizing. As John Dewey (1933) once wrote, “We do not learn from experience... we learn from reflecting on experience.” This is the essence of the above approach.

Finally, the results showed that the post-survey attitudes scores of primary and secondary preservice teachers did not show any statistical difference. This indicates that the introductory inclusion-centered course paired with field training effectively changed attitudes of both primary and secondary education preservice teachers toward inclusive education. This can be explained by the course's robustness and field experience that changed all participants' attitudes. Additionally, all the participants had relatively little teaching experience and did not go through difficult classroom experiences that may lead to significant differences in their attitudes.

The results of this study have implications for developing a curriculum for preservice teachers. Preservice education programs should include a sound theoretical component and an applied dimension where students are provided with opportunities to engage in meaningful contact with people with disabilities. Preservice teachers should also be taught ways to modify their “standard” teaching skills in ways that meet the needs of all learners within “inclusive frameworks” (Bayliss, 1998; Hassanein, 2015b). These programs need to be continuous, useful, and appealing. Furthermore, teachers should develop competency utilizing collaborative frameworks (Pugach and Blanton, 2009). These frameworks are based on the notion that collaboration represents a robust, systematic integration of general and special education across all aspects of the preservice curriculum that advances inclusion.

Additionally, the study's findings provide evidence that it is fundamental to improve the efforts of creating inclusion-centered curricula for preservice teachers. This is in line with the findings of Tournaki and Samuels (2016). They argued that since general and special educators share the responsibility of providing an appropriate education for all students in inclusive settings, teacher-preparation programs should focus on inclusive education preparation. This can be achieved through a curriculum that integrates meaningful fieldwork experiences in inclusive education settings. Additionally, a range of methods can be integrated into this curriculum. These include teacher-student interactions, co-teaching, peer mentoring, and opportunities to observe collaborative practices that facilitate inclusion (Swain et al., 2012).

Finally, researchers suggest that one of the most significant barriers to the development of inclusion is that most teachers lack the essential knowledge, skills, and attitudes to carry out this work (Forlin, 2004; Forlin and Chambers, 2011; Gaad and Almotairi, 2013; Hassanein et al., 2021). Therefore, we must prepare preservice teachers who have the confidence and the skills to provide appropriately differentiated instruction for all learners. All these suggestions and implications are supported by other research (e.g., Fullan and Hargreaves, 1992) that supports the practitioners in the early stages of implementing an educational reform that is not enough; continued support, real contact experience, and technical assistance must be provided.

### 4.1 Limitations

Nonetheless, the current study has some limitations that suggest caution in interpreting the results. First, the current study's findings, like most studies in this area (de Boer et al., 2011), are based on self-reports in survey research, as opposed to observations of teachers' instruction. Future research may consider using observations of teachers' instruction, as it could be an accurate representation of participants' behavior better than self-report surveys. Second, the data was collected upon completion of an intensive course on inclusive education, when these issues might be emphasized for these preservice teachers. Therefore, the progress of the participants' attitudes over more extended periods cannot be inferred. Additionally, this change in the participants' attitudes may be 'transitory' (Campbell et al., 2003) or 'temporary' (Tournaki and Samuels, 2016). These changes may not be maintained once these preservice teachers face challenging classroom experiences with children with a range of disabling conditions.

Therefore, further follow-up studies are required to assess the effect of the course on preservice teachers' attitudes toward inclusion after a follow-up period that could be one semester or by the end of the training program to check if the participants' attitudes progressed or regressed. Last, all participants were female students; findings drawn from this sample may not generalize to other populations. Future research should consider male samples for better comparisons across gender.

In conclusion, the study highlights the possibility of changing preservice teachers' attitudes toward inclusive education. The results suggest that the introductory inclusion-centered course combined with meaningful field experience may positively change preservice teachers' attitudes and develop their abilities and skills to facilitate inclusive education. Therefore, when planning inclusion-centered courses, teacher preparation programs must consider combining a meaningful field experience within an inclusionary setting. Finally, teacher-preparation programs should maintain their focus on inclusive education preparation.

## Declarations

### Author contribution statement

Elsayed Hassanein: Conceived and designed the experiments; Performed the experiments; Analyzed and interpreted the data; Wrote the paper.

Yousef Alshaboul: Conceived and designed the experiments; Analyzed and interpreted the data; Contributed reagents, materials, analysis tools or data.

Sayed Ibrahim: Conceived and designed the experiments; Contributed reagents, materials, analysis tools or data.

### Funding statement

The Open access funding is supported by Qatar National Library.

### Data availability statement

Data will be made available on request.

### Declaration of interests statement

The authors declare no conflict of interest.

### Additional information

No additional information is available for this paper.
